# Supplementary figures and images for: Horizontal Transfer and Evolutionary Profiles of Two Tc1/DD34E Transposons (ZB and SB) in Vertebrates
Source: Genes (Basel). 2022 Nov 29;13(12):2239. doi: 10.3390/genes13122239 (PMC9777934; doi:10.3390/genes13122239)

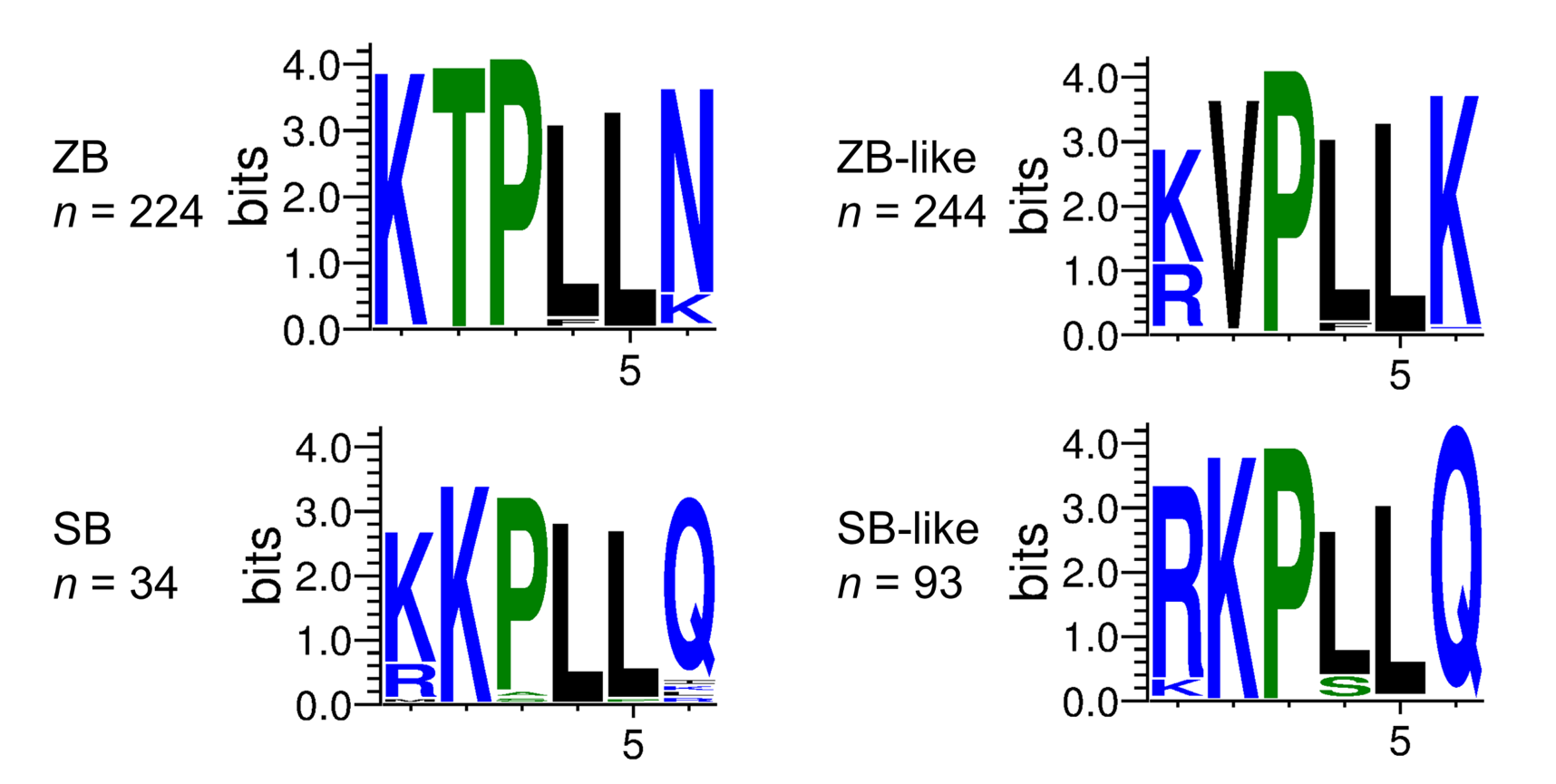

Supplement: Supplementary file 1 [file genes-13-02239-s001.zip › supplementary Figure S2.tif]

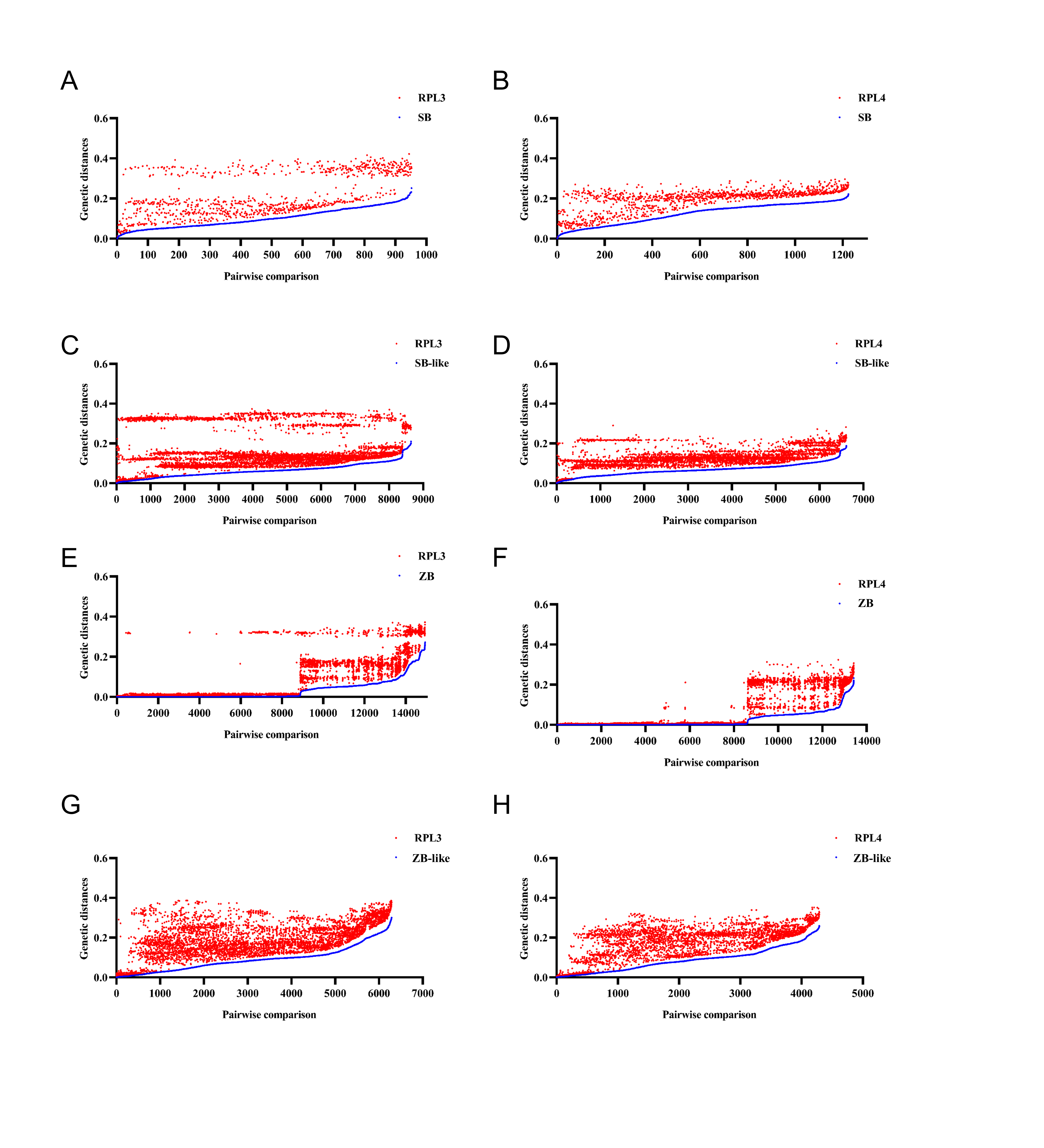

Supplement: Supplementary file 1 [file genes-13-02239-s001.zip › Supplementary Figure S3.tif]

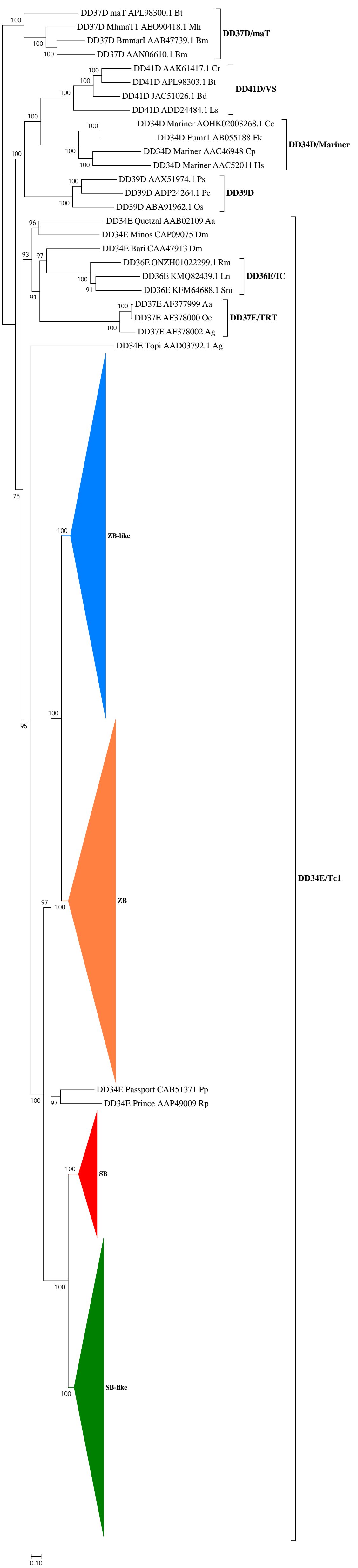

Supplement: Supplementary file 1 [file genes-13-02239-s001.zip › Supplementary Figure. S1-B.PDF]
