# Supplementary material for: Horizontal Transfer and Evolutionary Profiles of Two Tc1/DD34E Transposons (ZB and SB) in Vertebrates
Source: Genes (Basel). 2022 Nov 29;13(12):2239. doi: 10.3390/genes13122239 (PMC9777934; doi:10.3390/genes13122239)

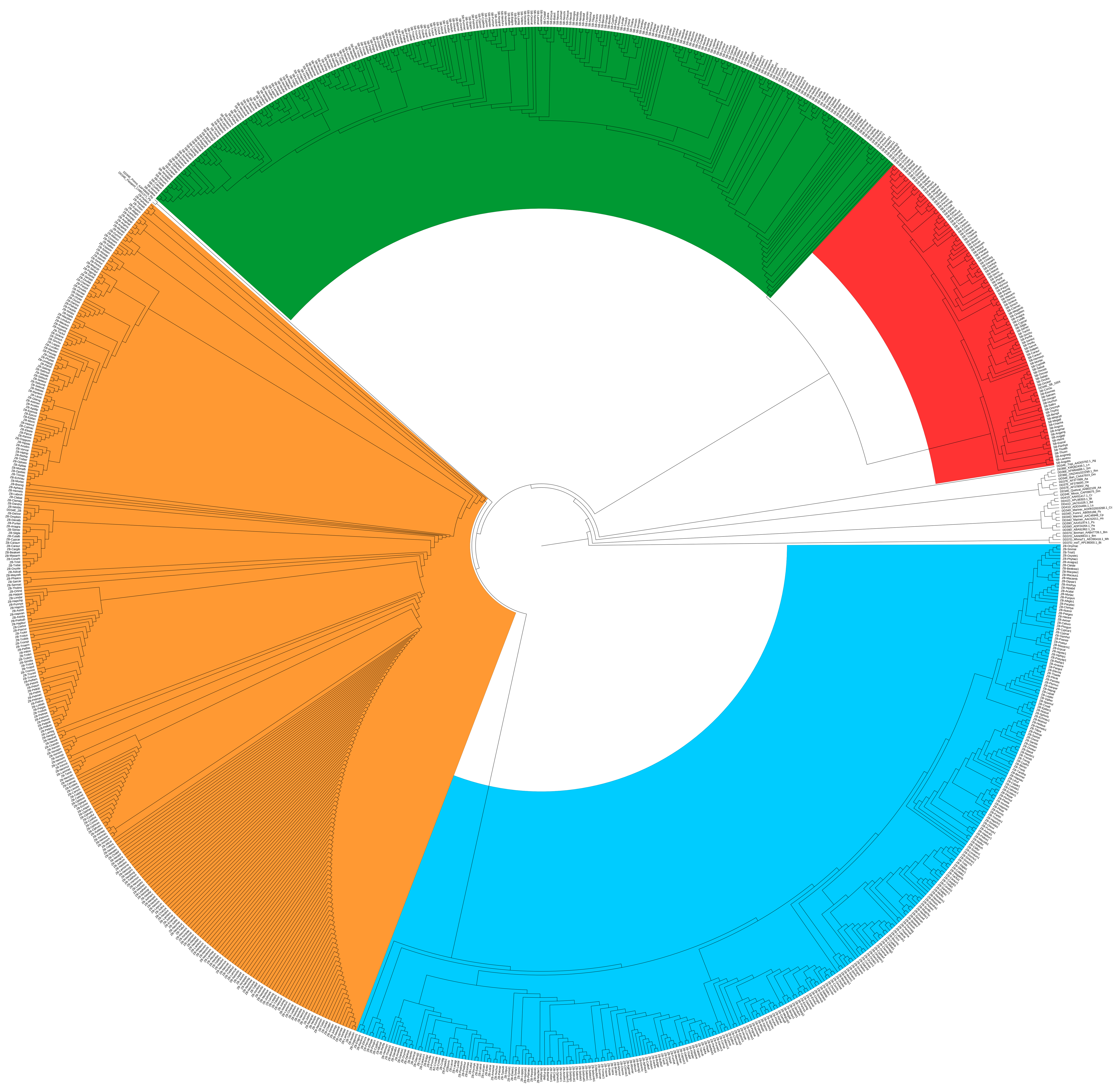

Supplement: Supplementary file 1 [file Supplementary Figure. S1-A.pdf › Supplementary Figure. S1-A.pdf]
